# The Perception of Effort as a Basis for Improving Physical Efficacy and Efficiency in Italian Military School Students

**DOI:** 10.3390/sports13040128

**Published:** 2025-04-21

**Authors:** Gabriele Signorini, Raffaele Scurati, Andrea Bosio, Maurizio Pizzoli, Angelo Pagano, Gaetano Raiola, Pietro Luigi Invernizzi

**Affiliations:** 1 Department of Biomedical Sciences for Health, Università degli Studi di Milano, 20133 Milan, Italy; gabriele.signorini@unimi.it (G.S.); maurizio.pizzoli@unimi.it (M.P.); angelopagano619@gmail.com (A.P.); pietro.invernizzi1@unimi.it (P.L.I.); 2 Human Performance Laboratory, Mapei Sport Research Centre, 21057 Olgiate Olona, Italy; andrea.bosio@mapeisport.it; 3 Research Centre of Physical Education and Exercise, Pegaso University, 80143 Napoli, Italy; gaetano.raiola@unipegaso.it

**Keywords:** circuit training, motivation, military admission test, physical education

## Abstract

Military schools primarily aim to prepare young people for the admission procedures of military academies. In this specific environment, the high overall load can generate burnout in cadets and the consequent failure to achieve scholastic and military objectives. The present study investigated how a training protocol based entirely on internal load and a reflective approach in a military-type school context affects participants’ physical efficacy, efficiency, and psychological outcomes. For this study, 63 cadets who were 17 years old from an Italian military school were recruited. Twenty-two of them were allocated into a control group (CG), twenty-one were allocated into a group exercising based on external load (EG), and twenty we allocated into a group exercising based on internal load (IG). All groups performed tests of physical efficacy (maximal tests) and physical efficiency (self-perception-based submaximal test) and answered psychological questionnaires to assess motivation, self-efficacy, and enjoyment. Group participants attended eight weeks of interventions in which physical education lessons were led as follows: the EG performed a circuit training at 50% of maximal repetitions, the IG performed a circuit training at value six on Borg’s scale, and the CG attended curricular physical education lessons. Tests were then repeated. The IG increased physical efficacy more than the EG and CG, while only the IG increased physical efficiency. The IG and EG improved in psychological variables more than the CG. Education in self-perception and self-regulation could help cadets better manage their psychophysical status, allowing them to reach the physical demands for academic admission.

## 1. Introduction

To carry out their duties, military personnel need good physical capacities [1,2] and to be trained in an integrated manner with military training [3] that can be useful for specific skills [4,5].

For these reasons, in many countries around the world, to access military careers at different levels (soldier, non-commissioned officer, officer), it is mandatory to pass a selection procedure that verifies not only the cultural grade and psychological aptitudes of the candidates but also, above all, their physical abilities through predetermined batteries of evaluation tests (physical efficacy) [6,7].

More specifically, a military career in Italy can begin at a young age by attending military schools, starting at 16 years old. These schools primarily aim to prepare young people for the admission procedures of military academies and, of course, to pass the aptitude tests required for access. Furthermore, since they are educational institutions, their curricula include all the educational aspects required by the school education system and, in regard to physical education, the so-called promotion of physical literacy. Physical literacy can be defined as the set of physical skills, motivation, confidence, knowledge, and understanding necessary to maintain an adequate level of general physical activity throughout the entire life of the individual [8,9].

In this regard, military school routines aspire to educate cadets on developing high conscientiousness to plan, achieve goals, and take on individual responsibility. In these training processes, the perception of self-efficacy is a relevant mediator. A greater sense of self-efficacy predisposes cadets to an effective personal adaptation (and of their own needs) to impact and modify the context in which they act (planning and achieving goals). Furthermore, this self-perception is positively correlated to increased specific skills in the military context [10,11]. Some studies have shown how the sense of self-efficacy can be increased through dedicated programs that aim to favor direct personal experiences, social persuasion, vicarious learning (through positive models), and psychological feedback (emotional states) [12].

In this perspective, self-regulatory learning and autoregulatory training are two specific approaches to consider. The first refers to student engagement during the learning process to improve and consolidate a specific task [13]. Its theoretical foundation relies on the social cognition model [14], which considers constructs such as self-efficacy, effort, self-monitoring [15], motivation [16], and metacognitive strategies [13]. In a military school environment, self-efficacy consists of a self-assessment of one’s skills and ability; effort describes the intensity of the involvement in task execution, as in training during physical education classes or extracurricular physical activities; self-monitoring helps the subject to address the perceived exertion necessary to manage the execution of specific training in the function of the tests for admissions to the military academy [15]; motivation represents the intensity of the desire to achieve specific psychophysical goals and is influenced by self-efficacy belief [16]; and metacognitive strategies are based on the awareness of one’s goals and the ability to monitor the learning process and reflect on the adequacy of one’s learning level in order to modify it, if necessary [13].

Niemiec and Ryan [17] demonstrated that self-regulation learning determines a high intrinsic motivation in subjects, characterized by pleasure, enjoyment, physical self-efficacy, and persistence in effort during physical education lessons. More specifically, enjoyment and physical self-efficacy represent the key factors that can promote motor and physical activity, allowing the individual to adhere to physical motor practices, thus contributing to the promotion of physical literacy [18,19].

Autoregulation training is a process of adaptation and measurement of physical efficacy based on acute and chronic fluctuations in strength performance, where performance includes the sum of factors affecting training (fatigue, fitness) and other factors complementary to training, such as readiness, sleep, nutrition, stress, psychological availability, willingness to train, and perceived available energy level [20,21]. Physical efficacy, that is, the ability to accomplish specific goals or standards, such as the physical military test for academies or maximal test evaluation, can also be pursued by building a more efficient movement and increasing the level of physical performance [22,23]. Physical efficiency (economy of movement) is the ability to perform physical exercises with the least effort possible. Due to the military profession’s highly demanding tasks (internal and external load), it is of high importance for cadets to increase the level of feedback and their consciousness about movement (as the RPE) and learn to perceive body effort signals during action. The ability to maintain a fair level of effort with a high amount of repetition represents a solid base for developing greater physical efficacy [22,23,24].

In the autoregulatory training approach, there are two possible manipulations of effort intensity and volume, both showing similar results: a subjective self-regulated one based on perceived effort (internal load) and an objective one based on external load (e.g., velocity-based training) [25]. Internal load can be an indicator reflecting the psychophysiological response of the body to requirements elicited by the external load. Therefore, the concept of internal load incorporates all the psychophysiological responses to physical and psychological tasks (often occurring during cadets’ routines). Moreover, perceived effort represents a modality to monitor internal load [26].

In particular, the subjective approach relies on effort perception strategies, where the number of prescribed sets and each set ends upon reaching a predetermined subjective rate of perceive exertion (RPE) level. It prevents some undesired circumstances of a more standardized approach, in which a pre-calculated load can result in inappropriate conditions for overtrained athletes or ones not responding positively and adequately to the training program. Therefore, a flexible program based on perceived exertion can be more effective than a traditional program for several reasons: overtraining, boredom, and lack of interest can be avoided, motivation can be restored, and loads can be modified based on the individual’s physiological and psychological readiness for a particular type of training [20,21].

In this context, physical education lessons are a type of specific training that, when based on a self-regulation methodological approach and on the ability to perceive psychophysical effort and fatigue, allows students to acquire greater self-awareness and self-management, and to learn to manage their energies according to the “real tasks” and the stressful load that military school orientation imposes. To the best of our knowledge, no studies have considered aspects of self-regulatory learning and autoregulatory training during physical education classes in military contexts, which represents the innovative potential benefits of the present study.

This class-randomized control trial aimed at investigating in a military-type school training context how a training protocol based entirely on subjective autoregulatory training (perception of effort) and on the principles of self-regulatory learning affects the physical efficacy (reaching physical prefixed goals), physical efficiency (performing with optimal physical effort while conserving energy), and psychological outcomes (bound to motivation enjoyment and self-efficacy). The results from this approach were also compared with similar training protocols based on external load (sets, repetitions calibrated on a maximum load) and the school’s standard physical education lessons (control group).

Significant improvements are expected in both intervention groups. However, we hypothesized that training based on internal load would obtain better results than training based on external load, particularly in efficiency tests based on perceived effort.

The research results will allow us to verify innovative methodologies for managing the training load and preparing students in a physical education context within a military-type formation. Furthermore, the intervention will provide the participating students with new opportunities for education in awareness and body management that will allow them to better control the psychophysical stress to which they are and will be subjected during their future military careers.

## 2. Materials and Methods

### 2.1. Participants

Sixty-three male students from the Teulié military school in Milan were recruited for this study. The sample size was calculated with G*Power software (v. 3.1.9.7), using a statistical power of 90%, alpha = 0.05, and an estimate of the effect size (f^2^) equal to 0.15. For this calculation, the multiple linear regression model was chosen (*t*-test—linear multiple regression—fixed model, single regression coefficient). Fifty-four participants were needed to satisfy the statistical power. The inclusion criterion required that participants attended at least 70% of the lessons planned for the experimental protocol. The exclusion criteria were the presence of injuries or other conditions that prevented the participant from actively taking part in physical education lessons. Participants were cadets of a military school, so they were used to performing rigid daily life behaviors and activities (as they lived inside the school) such as defined sleeping hours, mealtime, diet, and curricular and extracurricular activities (two hours twice a week, and quarterly schedules including fencing, horse riding, and athletics).

The study and data collection were carried out at the Teulié Military School in Milan, in collaboration with the physical education teachers and with the authorization of the Commander and Chief of Staff. Therefore, the study participants (the students at the school) benefited from the Teulié Military School’s insurance coverage. All participants and parents were given a detailed explanation of the study procedures and potential risks. They then provided written consent and were informed that they could withdraw from the study at any time. The study complied with the principles of the Declaration of Helsinki and received approval from the local University Ethics Committee (protocol code 44/23 of 18 April 2023).

### 2.2. Measures

Age, sex, and anthropometric measurements (weight, height, and BMI) were collected (Table 1).

Subsequently, tests were performed to evaluate physical efficiency (by RPE) and maximum physical capacity (physical efficacy), and some questionnaires were also administered to assess motivation, the enjoyment of physical activity, the sense of self-efficacy (psychological variables), and the amount of physical activity performed (weekly physical activity).

#### 2.2.1. Physical Efficacy Tests

Physical efficacy concerned two kinds of performances related to the following:

- Maximum circuit training [27] is a maximal test aimed at evaluating the physical capabilities (physical efficacy) of the participants on which to base the subsequent training sessions provided for by the experimental protocol. Participants are asked to perform six different maximal exercises for one minute each. During the work minute, the repetitions are counted, after which the perception of effort is evaluated to assess whether the effort made corresponds to the maximum perceived by the participant. At the end of the test, a complete recovery lasting 4 min is required before moving on to the next exercise. Exercises are performed in the following order: lower limb squats, push-ups, rope climbing, crunches, rope jumps, and pull-ups.
- Physical tests for military academy admission competitions [28] (physical efficacy) aim to verify the minimum physical preparation requirements and are included in the announcements for academy admission to the various Corps published in the official journal of the Italian Republic, whose corresponding evaluation scores are further indicated. Tests related to strength (push-ups, sit-ups, and pull-ups) and endurance (2000 m run) were considered.

#### 2.2.2. Physical Efficiency Test

The Cubo fitness test (CFT) [29] was used to assess the participants’ physical efficiency. It comprises five submaximal motor tests based on the perception of effort and pain performed on a multifunctional cube-shaped instrument (physical efficiency). It provides an index of motor efficiency (range 0–100), resulting from the scores achieved in the submaximal tests that specifically evaluate cardiorespiratory fitness [30], upper limb and abdominal strength [31], scapular-humeral mobility [32], and flexibility [33]. Prior to this, to administering the tests, the Total quality recovery scale (TQR) served to check that the psychophysical recovery status of participants was between au values from 13 (reasonable recovery) to 16 (good recovery), indicating that the level of recovery was satisfactory and not altering the perception of the effort [34].

#### 2.2.3. Psychological Questionnaires

Matthew’s motivational questionnaire [35] is a questionnaire that assesses the motivation to perform a specific task. The questionnaire is composed of two scales such as “achievement motivation” and “intrinsic motivation”, each composed of 7 items to which the participant must give a rating from 0 to 4 on a 5-point Likert scale, corresponding to “not at all”, “a little”, “moderately”, “quite a bit”, or “extremely”. The scores obtained on both scales can vary from 0 to 28.

The physical activity enjoyment scale (PACES) [36] is a 16-item questionnaire that uses the 5 points of the Likert scale (1: completely disagree, 2: disagree, 3: uncertain, 4: agree, 5: completely agree) to assess enjoyment. It is composed of two subscales: PACES_P, which measures positive feelings, and PACES_N, which measures negative feelings.

The physical self-efficacy scale (PSES) [37] assesses the self-perception of one’s physical efficiency in motor skills, which is considered a primary motivational factor for voluntary participation in any physical and sporting activity.

The general self-efficacy scale is a 10-item questionnaire [38] for the self-assessment of self-efficacy, expressed by a score between 10 and 40 points (high score = greater self-efficacy).

#### 2.2.4. Weekly Physical Activity

The physical activity questionnaire for adolescents (PAQ-A) [39] aims to determine the level of physical activity of the last seven days, including sports, recreational activities, dance, climbing, cycling, and unstructured activities. Low scores (1 to 2.33) correspond to a low PAL, medium scores indicate a moderate PAL (2.34 to 3.66), and high scores (3.67 to 5.00) imply a high PAL. The PAQ-A gave information about the frequency of physical activity as all cadets recruited performed the same typology of physical activity composed by two sessions per week of Physical Education classes and extracurricular sports proposed by the academy (athletics, fencing and horse riding), four hours per week.

### 2.3. Procedure

As the study was conducted in a military school, a class-randomized controlled trial was performed [40]. All measurements were performed in the school gym during physical education lessons. The repeatability of the motor tests was also performed using the test–retest system to calculate the intraclass correlation of the coefficient of variation. The intervention lasted 8 weeks and was administered by the same PE teacher with the help of two researchers. Participants were divided into three groups based on their classes to obtain three homogeneous groups regarding number, age, and sex. The three groups were defined as follows:

- External load experimental group (EG);
- Internal load experimental group based on the perception of effort (IG);
- Control group (CG).

Participants were familiarized to the training and testing procedures for two weeks. Afterwards, they followed the 8 weeks of intervention within the curricular physical education lessons provided by their school program (2 lessons per week of 60 minutes each). The content of the lessons was diversified based on the group to which they belonged. The circuit training of the experimental groups was performed once a week in one of the two scheduled curricular lessons. The second weekly lesson was common to all three groups (EG, IG, and CG) and carried out as required by the school program.

The training sessions based on circuit training were diversified based on the group to which they belonged:

- The EG performed all the circuit exercises using 50% of the repetitions performed in a previous maximum test. No rest was provided between the exercises. The circuit was repeated 3 times per session. During the execution of the circuit, the time and the perception of effort at the end of each exercise were recorded.
- The IG performed all the circuit exercises until they reached a perceived effort value of 6 (strong) with respect to a previous maximal test, assessed through the Borg CR-10 scale [41]. This procedure required a strong involvement of attention and constant reflection on the adaptations/modifications to which the external load must be arranged in relation to one’s psychophysical state. No breaks were planned between the exercises. The circuit was repeated 3 times per session. During the execution of the circuit, the execution time at the end of the circuit and the number of repetitions at the end of each exercise were also recorded. The IG participants were not informed about the results achieved during the maximal tests or the number of repetitions performed at the end of each exercise.
- The CG performed the regular curricular sport-based physical education lessons provided by the school.

All participants were also given a training diary in which the characteristics of the extracurricular training sessions were reported, with the execution time and the perception of effort detected at the end of the activity. All participants performed the same type of extracurricular activity and had to record the number of presences. The lessons and the testing sessions were further videorecorded to successively check the procedures.

### 2.4. Statistical Analysis

Descriptive statistics (means and standard deviations) were performed. The normal distribution of data was tested using the Shapiro–Wilk test. Where necessary (e.g., in motor tests), test–retest reliability was assessed in absolute and relative terms by calculating the coefficient of variation and the intraclass correlation coefficient. The differences between the IG, EG, and CG delta scores (post–pre) were assessed using a one-way ANOVA and Bonferroni correction post hoc test. In the case of non-parametric data, the Mann–Whitney U test was performed instead of one-way ANOVA. The significance level was set at 0.05. Effect sizes were evaluated using the Eta squared with the following cut-offs: 0.01–0.06 (small effect), 0.06–0.14 (moderate effect), and ≥0.14 (large effect).

## 3. Results

### 3.1. Tests’ Reliability

Maximal tests and the Cubo fitness test results were reliable, with ICC values higher than 0.70. Results are reported in Table 2.

### 3.2. Physical Efficacy

The delta analysis of maximal tests of the circuit training revealed that the IG obtained higher delta scores than the CG for push-ups (*p* = 0.010; Post hoc: *p* = 0.009), crunches (*p* = 0.034; Post hoc: *p* = 0.030), and the rope climb (*p* = 0.002; Post hoc: *p* = 0.001). Similarly, the EG obtained higher delta scores than the CG in the rope climb (*p* = 0.002; Post hoc: *p* = 0.006).

**Figure 1:**
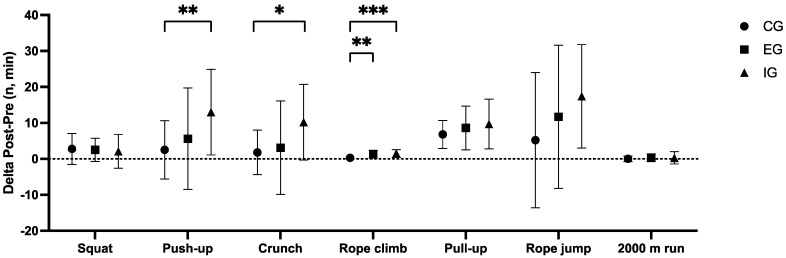
Comparisons of Deltas of maximal tests (physical efficacy) results depending on different interventions. CG = control group; EG = external load group; IG = internal load group. Significant differences: * = *p* < 0.05, ** = *p* < 0.01, *** = *p* < 0.001.

In contrast, no differences were found in the 2000 m run test. Results are reported in Table 3 and Figure 1.

### 3.3. Physical Efficiency (Cubo Fitness Test)

**Figure 2:**
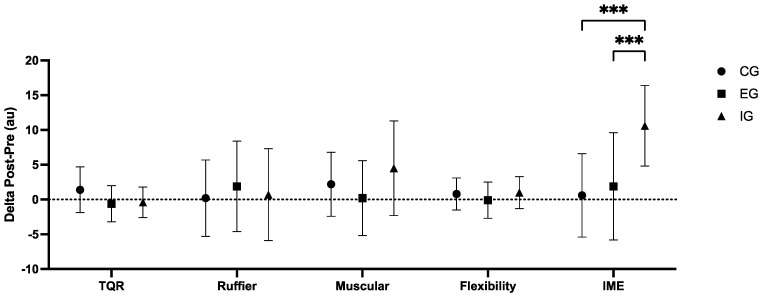
Comparisons of the Cubo fitness test (physical efficacy) results depending on different interventions. CG = control group; EG = external load group; IG = internal load group; TQR = total quality recovery; IME = index of motor efficiency. Significant differences: *** = *p* < 0.001.

In the CFT delta analysis, significant differences (*p* < 0.001) were found in the final score (IME). Post hoc testing revealed that the IG had higher delta scores than the CG (*p* < 0.001) and EG (*p* < 0.001). CFT results are reported in Table 4, while the comparisons of deltas (post-pre) are displayed in Figure 2.

### 3.4. Psychological Outcomes

**Figure 3:**
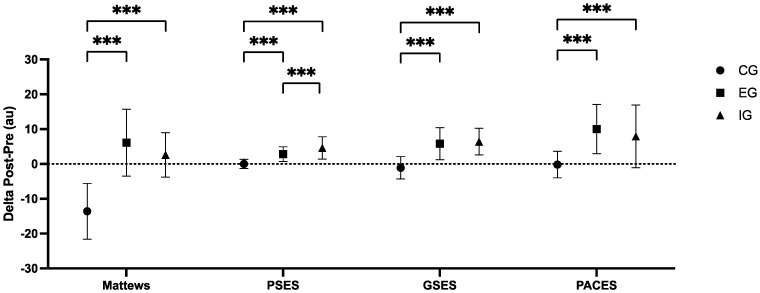
Comparisons of Psychological questionnaire results depending on different interventions. CG = control group; EG = external load group; IG = internal load group; PSES = physical self-efficacy scale; GSES = general self-efficacy scale; PACES = physical activity enjoyment scale. Significant differences: *** = *p* < 0.001.

The results are detailed in Table 5 and Figure 3.

### 3.5. Amount of Physical Activity

Delta analysis found no difference in the amount of physical activity. The PAQ-A results are displayed in Table 6.

## 4. Discussion

The intervention investigated how a training protocol based on subjective autoregulatory training (perception of effort) and on the principles of self-regulatory learning in a military-type school training context affects the participants’ physical efficacy, physical efficiency, psychological components, and the results of the physical test for admission to military academies. The analysis also compared the results obtained with similar training protocols based on external load and a control group that carried out the school’s regular physical and sports education lessons.

The intervention improved the conditional capacities related to the maximal and sub-maximal tests. After the intervention, the IG reported better physical efficacy than the control group. In particular, the IG resulted in higher changes in the maximal push-ups, sit-ups, and rope climbing tests. This result is not surprising, as several studies demonstrate the effectiveness of circuit training in improving muscle strength, positively influencing tests with maximum repetitions [42,43,44]. Nevertheless, the most interesting result is that the IG improved in more tests than the EG. As hypothesized, the results could originate from the better management of the training load. Indeed, basing the number of repetitions to be performed in circuit training on one’s psychophysical state allows one to obtain a training stress that is adequate to the daily management capacity of the body [45]. Hence, training daily on perceiving the body signals (perceived exertion) indicating the actual effort state could allow cadets to maintain an adequate number of repetitions (e.g., 8 out of 12) depending on the body’s psychophysical state (e.g., if normally a cadet perceived vigorous effort after 12 repetitions, when fatigued after a long school day, they could have the same perception after only 8 repetitions). Military school cadets are subjected to considerable daily workloads due to the amount of study and specific training [46,47]. A training load considering these variables could have allowed for a more favorable workload/recovery ratio, avoiding overtraining or poor recovery [42,45].

Regarding the maximal 2000 m test, no differences were found between groups. Even if some studies have evidenced the efficacy of circuit training in increasing anaerobic endurance, the training frequency (2 h of lessons per week for 8 weeks, of which only one hour was devoted to circuit training) could result in a workload that is too low to decrease this test’s time. The literature confirms that training three times per week for at least 8 weeks can be considered sufficient to increase the results in anaerobic endurance tests [48,49]. Conversely, the time dedicated to the intervention was considered adequate by the literature to gain effective results in maximum circuit training [42,44], but more data are required to assess whether they are maintained through time.

The Cubo fitness test results showed that the IG significantly had a higher delta score (physical efficiency score) than those of the CG and EG. This result, as hypothesized, may have depended on the dual effect of the improvement in the maximal tests and the greater sensitivity of the IG to perceive and regulate their effort. The first effect is known in the literature: an increase in conditional capacities corresponds to a decrease in the perception of effort for the same intensity of exercise or (in this specific case) for the same number of repetitions [48]. Consequently, with the same perception, the number of repetitions performed increases. The second effect, on the other hand, depends on the participant’s familiarity with perceiving and regulating the effort based solely on perceived exertion. Indeed, while the EG had to limit themselves to perform the sessions at defined repetitions, the IG had to adjust the effort, session by session, by adapting themselves to the actual psychophysical condition [50,51].

As an individual’s effort perception is a continuous decision-making process about whether to stop investing resources towards a task, increasing physical efficiency (i.e., movement economy) could be the clue for better self-management [52]. Cadets regularly face individual responsibilities determined by recurrent challenging daily tasks [53]. Hence, the consciousness about personal status and psychophysical availability constitute an indispensable tool to reach the prefixed goals of respecting and preserving mind and body integrity in different situations [54].

The cadets from the Teulié school who took part in the experiments as part of the IG and EG reported positive effects of the intervention regarding the values of motivation, enjoyment, physical self-efficacy, and general self-efficacy. In particular, the IG and EG obtained similar results, both of which were better than the control. The increases in motivation in the intervention groups compared to the control group depend on the nature of the proposed activity. The circuit training designed for the intervention included exercises that were similar and functional to the physical tests proposed in the entrance tests for military academy admission, thus representing a useful path to achieving an important training goal that is common to both groups. This is particularly relevant as studies discussed by Caforio (2018) [55] highlight how the motivation of cadets is crucial to the development of their future professional careers. Moreover, as intrinsic motivation is also associated with an appreciation of the activity one intends to carry out [56], this can also explain why the enjoyment of the activity and the motivation of the IG and EG were higher compared to the CG.

Also, the increase in the self-efficacy values of the IG and EG could be closely linked to increased motivation [57]. The literature shows that for military cadets, the increase in motivation (*I want to do…*) is fundamental in professional growth [58] and is linked to the perception of being able to carry out a pre-established task (*I can do…*). The IG and EG could have perceived more than the CG (and the IG even more so than the EG in the physical self-efficacy) the possibility of success in the task because the personalization of the goals and the consistency in the request could, therefore, have led the participants to perceive the proposals as a successful task, increasing their sense of physical self-efficacy (*I can finish the exercises*) and overall efficacy (*I completed the set tasks*) [12,59]. Focusing on physical self-efficacy, the IG could have benefited more from this effect also because the group experienced success in each lesson [60,61]. Indeed, when the IG experienced the circuit training, cadets completed the task according to their psychophysical status, avoiding the perception of excessive effort depending on daily psychological and body condition (as they did not have to reach a prefixed number of repetitions conversely to the EG). Hence, the perception of higher fatigue than usual to perform the same exercise could have lowered the results of physical self-efficacy of the EG [62].

A limitation of this study concerns the relatively short duration of the training period, which lasted eight weeks. The curricular and service commitments of the cadets involved in the study made it impossible to extend the intervention further. However, previous studies, both under conditions of external load control and internal load control, have observed sensible effects on physical and psychological variables even with protocols of comparable durations [42,44]. Based on this, the experimental period can be considered adequate, although future studies could extend the duration of the protocol.

Another limitation is that the tool used for submaximal assessments, the Cubo fitness test, has not yet been validated in the military context. However, there is previous evidence that the tool is reliable in its application and it has been successfully used in contexts different from that of university students in which it was originally validated [63]. A future validation in the specific context of military high school students would be desirable.

## 5. Conclusions

**Figure 4:**
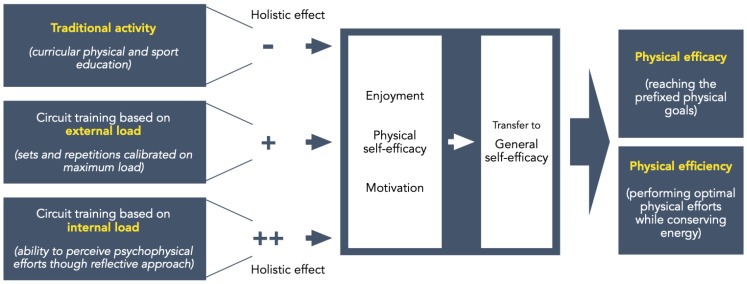
The framework depicting features and holistic effects of the approaches used in the present study (curricular activities, circuit training based on external load, and circuit training based on internal load). Holistic effects in the specific military context were trivial (−), partially appropriate (+), or appropriate (++).

In conclusion, circuit training lessons in military school during physical education classes can produce higher physical efficacy, physical efficiency, motivation, a sense of self-efficacy, and enjoyment when oriented to self-perception, helping to generate autonomy and competence. Moreover, the education on perceived exertion could guide cadets toward better daily self-management during physical tasks as it considers their actual psychophysical status. Figure 4 synthesizes in a framework the effects of the three intervention approaches administered in the present study (curricular activities, circuit training based on external load, and circuit training based on internal load) and their efficacy in improving military cadets’ psychological and psychophysical capacity.

## Figures and Tables

**Table 1:** Participants’ anthropometric data.

	Curricular Activities (CG)	External Load (EG)	Internal Load (IG)
n	22	21	20
Age (years)	17.3 ± 0.8	17.1 ± 0.9	17.1 ± 0.7
Height (m)	1.70 ± 0.08	1.73 ± 0.07	1.67 ± 0.10
Weight (Kg)	67.00 ± 8.37	67.64 ± 7.57	66.00 ± 10.08
BMI (Kg/m^2^)	23.7 ± 2.1	22.7 ± 1.9	23.5 ± 1.9

Curricular activities = control group.

**Table 2:** Tests’ reliability.

	Test	ICC Value	*p*-Value
Circuit Training	Squat	0.70	<0.001
	Push-up	0.80	<0.001
	Crunch	0.88	<0.001
	Rope climb	0.88	<0.001
	Pull-up	0.98	<0.001
	Rope Jump	0.89	<0.001
	2000 m run	0.70	<0.001
Cubo Fitness Test	Ruffier	0.70	<0.001
	Muscular	0.85	<0.001
	Flexibility	0.90	<0.001
	IME	0.78	<0.001

**Table 3:** Results of maximal tests (physical efficacy).

Exercise	Group	Pre	Post	Δ (Post-Pre)	Effect Size (η^2^)
Squat (n)	CG	26.5 ± 4.7	30.1 ± 4.4	2.8 ± 4.3	−0.052
	EG	30.7 ± 3.4	33.5 ± 5.2	2.5 ± 3.2	
	IG	30.9 ± 4.6	33.0 ± 5.2	2.1 ± 4.7	
Push-Up (n)	CG	33.9 ± 11.9	36.9 ± 10.6	2.5 ± 8.1	0.124
	EG	41.4 ± 11.4	46.6 ± 10.4	5.6 ± 14.1	
	IG	36.3 ± 10.1	49.3 ± 9.8	13.0 ± 11.9 #	
Crunch (n)	CG	43.6 ± 5.7	45.9 ± 6.1	1.8 ± 6.2	0.113
	EG	47.4 ± 9.6	50.8 ± 9.7	3.1 ± 13.0	
	IG	45.9 ± 9.4	56.1 ± 7.7	10.2 ± 10.5 #	
Rope climb (n)	CG	1.5 ± 1.1	1.7 ± 1.3	0.3 ± 0.8	0.225
	EG	1.4 ± 1.2	2.7 ± 1.5	1.3 ± 1.1 #	
	IG	1.8 ± 1.1	3.3 ± 0.9	1.5 ± 1.1 #	
Pull-Up (n)	CG	7.2 ± 4.7	14.5 ± 6.2	6.8 ± 3.9	0.079
	EG	7.4 ± 5.4	16.2 ± 9.9	8.6 ± 6.1	
	IG	10.9 ± 4.1	20.6 ± 7.6	9.7 ± 6.9	
Rope jump (n)	CG	112.7 ± 36.2	127.8 ± 25.4	5.2 ± 18.8	0.080
	EG	118.9 ± 25.6	130.7 ± 21.6	11.7 ± 19.9	
	IG	112 ± 27.9	129.4 ± 27.2	17.4 ± 14.4	
2000 m run (min)	CG	8.5 ± 1.1	8.5 ± 1.1	0.0 ± 0.8	0.081
	EG	8.2 ± 1.0	8.4 ± 1.6	0.3 ± 1.1	
	IG	7.7 ± 0.5	8.0 ± 1.6	0.3 ± 1.7	

CG = control group; EG = external load group; IG = internal load group; # = different than CG.

**Table 4:** Results of the Cubo fitness fest (physical efficiency).

Variable	Group	Pre	Post	Δ (Post-Pre)	Effect Size (η^2^)
TQR (au)	CG	13.5 ± 1.8	15.0 ± 2.9	1.4 ± 3.3	0.013
	EG	14.6 ± 1.9	14.0 ± 2.4	−0.6 ± 2.6	
	IG	14.5 ± 1.9	14.0 ± 2.9	−0.4 ± 2.2	
Ruffier (au)	CG	21.7 ± 5.1	21.6 ± 6.0	0.2 ± 5.5	0.016
	EG	21.0 ± 6.1	22.9 ± 5.4	1.9 ± 6.5	
	IG	23.8 ± 5.1	24.5 ± 6.4	0.7 ± 6.6	
Muscular (au)	CG	16.0 ± 6.2	17.8 ± 6.7	2.2 ± 4.6	0.093
	EG	20.5 ± 6.5	20.7 ± 5.5	0.2 ± 5.4	
	IG	16.6 ± 4.8	21.1 ± 6.4	4.5 ± 6.8	
Flexibility (au)	CG	8.3 ± 3.6	9.2 ± 4.2	0.8 ± 2.3	0.029
	EG	7.6 ± 3.2	7.5 ± 3.3	−0.1 ± 2.6	
	IG	7.8 ± 3.3	8.8 ± 4.2	1.0 ± 2.3	
IME (au)	CG	53.0 ± 9.6	53.0 ± 9.3	0.6 ± 6.0	0.394
	EG	52.1 ± 9.0	54.0 ± 8.1	1.9 ± 7.7	
	IG	48.8 ± 8.8	59.4 ± 6.9	10.6 ± 5.8 # §	

CG = control group; EG = external load group; IG = internal load group; TQR = total quality recovery; IME = index of motor efficiency; # = different than CG; § = different than EG.

**Table 5:** Psychological questionnaires results.

Variable	Group	Pre	Post	Δ (Post-Pre)	Effect Size (η^2^)
Matthews (au)	CG	39.4 ± 6.2	25.7 ± 4.7	−13.6 ± 8.0	0.562
	EG	37.2 ± 9.1	43.3 ± 2.9	6.1 ± 9.6 #	
	IG	40.4 ± 4.0	43.0 ± 4.6	2.6 ± 6.4 #	
PSES (au)	CG	14.7 ± 4.2	14.7 ± 3.9	0.0 ± 1.3	0.492
	EG	15.5 ± 4.7	18.3 ± 4.4	2.8 ± 2.1 #	
	IG	15.6 ± 4.6	20.2 ± 3.4	4.6 ± 3.2 # §	
GSES (au)	CG	30.1 ± 8.2	29.0 ± 7.3	−1.1 ± 3.2	0.467
	EG	27.3 ± 9.2	33.1 ± 8.0	5.8 ± 4.6 #	
	IG	31.4 ± 8.2	37.8 ± 6.3	6.4 ± 3.8 #	
PACES (au)	CG	50.6 ± 12.2	50.4 ± 11.6	−0.2 ± 3.8	0.456
	EG	51.2 ± 15.7	61.2 ± 12.0	10.0 ± 7.1 #	
	IG	52.1 ± 15.2	60.1 ± 9.2	7.9 ± 9.0 #	

CG = control group; EG = external load group; IG = internal load group; PSES = physical self-efficacy scale; GSES = general self-efficacy scale; PACES = physical activity enjoyment scale. # = different than CG; § = different than EG.

**Table 6:** Amount of physical activity.

Variable	Group	Pre	Post	Δ (Post-Pre)	Effect Size (η^2^)
PAQ-A (au)	CG	3.5 ± 0.4	3.6 ± 0.6	0.1 ± 0.8	0.009
	EG	3.3 ± 0.4	3.5 ± 0.3	0.2 ± 0.6	
	IG	3.6 ± 0.9	3.6 ± 0.6	0.0 ± 1.2	

CG = control group; EG = external load group; IG = internal load grou; PAQ-A = physical activity questionnaire for adolescents.

## Data Availability

The data supporting the main findings of this study are available on reasonable request with access granted to researchers meeting the criteria for access to confidential data. The data repository is Zenodo, at https://doi.org/10.5281/zenodo.14978543 (URL accessed on 6 March 2025).

## Abbreviations

The following abbreviations are used in this manuscript:

RCT	Randomized controlled trial
CG	Control group
EG	External load group
IG	Internal load group
CFT	Cubo fitness test
IME	Index of motor efficiency
PAQ-A	Physical activity questionnaire for adolescents
PACES	Physical activity enjoyment scale
PSES	Physical self-efficacy scale
GSES	General self-efficacy scale
